# TGFβ signaling regulates lipogenesis in human sebaceous glands cells

**DOI:** 10.1186/1471-5945-13-2

**Published:** 2013-01-23

**Authors:** Adrian J McNairn, Yanne Doucet, Julien Demaude, Marion Brusadelli, Christopher B Gordon, Armando Uribe-Rivera, Paul F Lambert, Charbel Bouez, Lionel Breton, Géraldine Guasch

**Affiliations:** 1 Division of Developmental Biology, Cincinnati Children’s Hospital Medical Center, 3333 Burnet Avenue, Cincinnati, OH, 45229, USA; 2 Current Address: Department of Biomedical Sciences, College of Veterinary Medicine, Cornell University, Ithaca, NY, 14853, USA; 3 Current Address: Department of Dermatology, Columbia University, College of Physicians and Surgeons, New York, NY, 10032, USA; 4 L’OREAL Research & Innovation, 90 rue du General Roguet, CLICHY, 92583, FRANCE; 5 Division of Plastic Surgery, Children’s Hospital Medical Center, 3333 Burnet Avenue, Cincinnati, OH, 45229, USA; 6 University of Wisconsin School of Medicine and Public Health, Madison, WI, USA

**Keywords:** Human Sebaceous gland cells, Sebocytes, TGFβ signaling, Cell differentiation, Proliferation, Lipogenesis, Skin appendages

## Abstract

**Background:**

Sebaceous glands are components of the skin essential for its normal lubrication by the production of sebum. This contributes to skin health and more importantly is crucial for the skin barrier function. A mechanistic understanding of sebaceous gland cells growth and differentiation has lagged behind that for keratinocytes, partly because of a lack of an in vitro model that can be used for experimental manipulation.

**Methods:**

We have developed an in vitro culture model to isolate and grow primary human sebocytes without transformation that display functional characteristics of sebocytes. We used this novel method to probe the effect of Transforming Growth Factor β (TGFβ) signaling on sebocyte differentiation, by examining the expression of genes involved in lipogenesis upon treatment with TGFβ1. We also repressed TGFβ signaling through knockdown of the *TGFβ Receptor II* to address if the effect of TGFβ activation is mediated via canonical Smad signal transduction.

**Results:**

We find that activation of the TGFβ signaling pathway is necessary and sufficient for maintaining sebocytes in an undifferentiated state. The presence of TGFβ ligand triggered decreased expression in genes required for the production of characteristics sebaceous lipids and for sebocyte differentiation such as *FADS2* and *PPARγ,* thereby decreasing lipid accumulation through the TGFβ RII-Smad2 dependent pathway.

**Conclusion:**

TGFβ signaling plays an essential role in sebaceous gland regulation by maintaining sebocytes in an undifferentiated state. This data was generated using a novel method for human sebocyte culture, which is likely to prove generally useful in investigations of sebaceous gland growth and differentiation. These findings open a new paradigm in human skin biology with important implications for skin therapies.

## Background

In humans, sebaceous glands associated with hair follicles are distributed throughout all the skin and found in greatest abundance on the face and scalp and are absent from the palms and soles [1]. Sebaceous glands can also form independently from the hair follicle and form specialized glands such as Meibomian glands of the eyelid, ectopic sebaceous gland of the glans penis [2] and Fordyce’s spots of the oral epithelium. Sebaceous glands are microscopic glands which secrete an oily substance (sebum) in the hair follicles to lubricate the skin and hair of animals [3]. Their function within the epidermis is to prevent the skin from dehydration and protect the body against infections and physical, chemical and thermal assaults of the environment. The main components of human sebum are triglycerides and fatty acids (57.5%), wax esters (26%), and squalene (12%) [4]. The production of sebum is regulated throughout life, and decreases dramatically with age [5]. This is associated with increased dryness and fragility of the skin. Moreover, several human diseases, such as acne vulgaris, atopic dermatitis, seborrheic dermatitis and primary cicatricial alopecia are thought to be associated with deregulation of the sebaceous glands [4,6,7].

There is a crucial interdependency of sebaceous glands with hair follicles and epidermis as sebocyte dysfunction results in degeneration of hair follicle structures and a defective skin barrier [7,8]. This is illustrated in the *asebia* mutant mouse, which lacks the SCD1 enzyme that desaturates fatty acids. This mutant displays rudimentary sebaceous glands and alteration in the profile of skin surface lipids leading to chronic inflammatory reactions, alopecia and dermal scarring [8].

Successful growth of primary human cells often constitutes a breakthrough in a specific area of human biology with important clinical implications. Tissue stem cells such as those of the blood and the epidermis have already been successfully used in clinics for decades [9,10]. In particular, epidermal cells (keratinocytes) can be cultured in vitro and can be efficiently manipulated to form a three dimensional epidermis [11,12]. Despite these advancements, the successful methods for culturing human primary sebocytes without the use of mouse feeder layers are not established. Selective cultivation of human sebocytes has been attempted in the past using mitomycin-treated 3T3 feeder layers by covering the microdissected sebaceous gland explant with glass slides but primary sebocytes survived only two passages after which they underwent differentiation [13]. Human sebaceous gland cell lines have been established in the past from adult human facial skin and periauricular area [14-17], but their immortalization with Simian virus-40 large T antigen or HPV16/E6E7 genes, which bypass the p53 and retinoblastoma protein mediated restriction point, results in cellular transformation that has limited their use for analyzing their cell cycle and differentiation regulation. Here, we culture human primary sebocytes using a novel method, which can in the future, be incorporated into skin reconstructs and provide a basis for understanding the molecular pathways which regulate human sebaceous gland biology.

A potential candidate for human sebocyte regulation suggested by several lines of evidence is Transforming Growth Factor β (TGFβ) [18,19] but the lack of primary human cultures has impaired an in-depth investigation of the molecular mechanism whereby TGF β signaling controls sebaceous gland differentiation. The TGF β pathway is ubiquitous and involved in the control of growth and differentiation of multiple cell and tissue types. The two major receptors of the TGFβ signaling pathway, TGFβ Receptor I (TGFβ RI) and TGFβ Receptor II (TGFβ RII), are expressed in mouse sebaceous glands [20,21]. In human and mouse epithelial cell lines, TGFβ acts as a potent inhibitor of proliferation mediated at least in part via down-regulation of c-Myc expression [22,23]. Intriguingly, c-Myc overexpression in a mouse model induces an increase in sebaceous gland size due to activation of sebocyte differentiation at the expense of hair differentiation [16,24]. Moreover, disruption of epidermal Smad4, the common mediator of TGFβ signaling, leads to hyperplasia of inter-follicular epidermis, hair follicle, and sebaceous glands through c-Myc upregulation [25].

To determine the effect of TGFβ signaling on sebocyte differentiation, we investigated the effect of TGFβ ligands on the primary human sebocytes we established using a novel culture system and skin samples from pediatric donors.

## Results

### Primary sebocytes established from pediatric donors express markers of sebaceous gland differentiation

**Figure 1:**
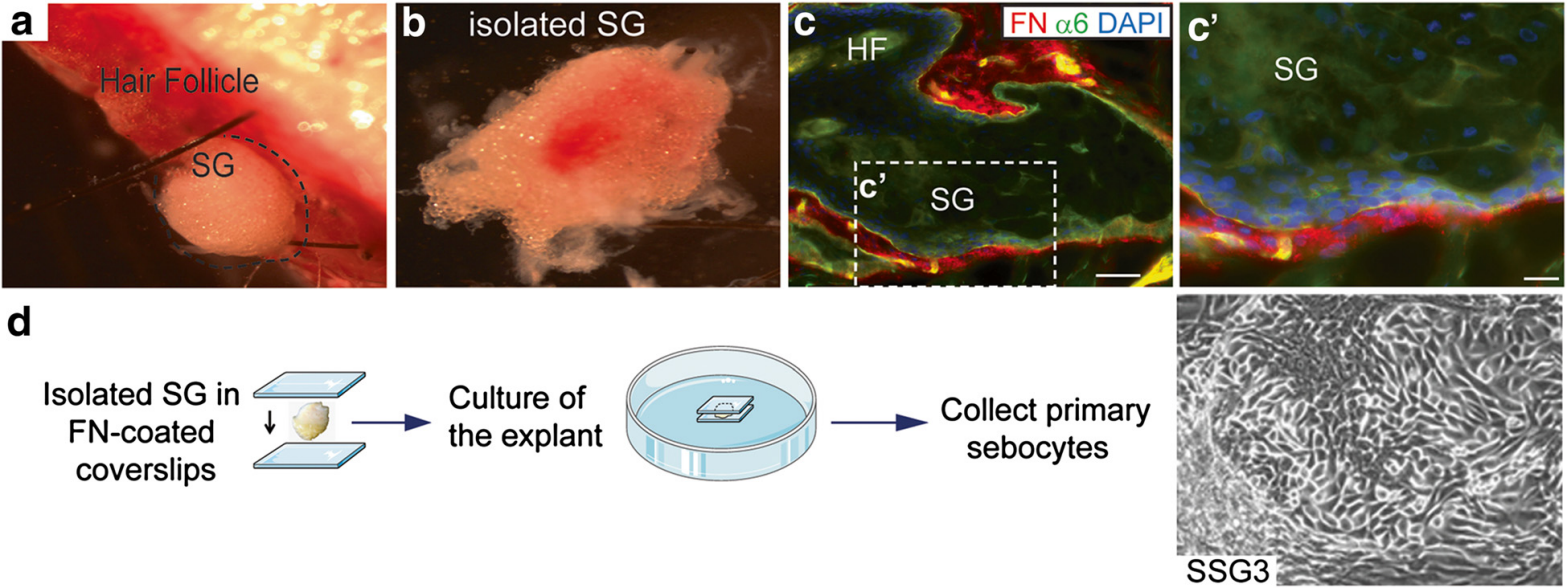
**Fibronectin mimics the microenvironment and allows sebocytes to grow in vitro*.*** (**a**) Scalp sample (9 months old) before microdissection. (**b**) Isolated sebaceous gland. **(c)** Immunofluorescence staining on OCT sections of human scalp tissue showed that fibronectin (in red) is expressed in the extracellular matrix surrounding the sebaceous gland. α6-integrin (in green) marked the basal layer of the gland. Boxed area is magnified and shown to **(c’)**. Scale bars, 20 μm (**c**, **c’**). (**d**) Schematic of new method to isolate and cultivate sebocytes. Scalp explants were placed between coverslips coated with fibronectin. Sebaceous gland cells SSG3 growing out of the explant (100x magnification). Abbreviations: SG, Sebaceous Gland, HF, Hair Follicle, FN, Fibronectin.

To determine the pathways that regulate primary human sebocytes growth and differentiation, we developed a novel culture method by mimicking the microenvironment of the sebaceous glands in vitro. Skin explants from donors ranging from 9 months to 12 years of age were microdissected (Figure 1a-b) and the sebaceous glands were placed between fibronectin-coated glass coverslips to reproduce an in vivo environment (Figure 1c-d). Using this technique, primary sebocyte cultures were derived from eight donors representing four skin tissue types: five scalp, one breast, one chest, and one face sample. While this technique enabled us to continually passage sebocytes beyond 15 passages, all experiments were performed on passage 2 and later passages (3 to 5) without the use of extracellular matrix or supporting irradiated fibroblasts.

**Figure 2:**
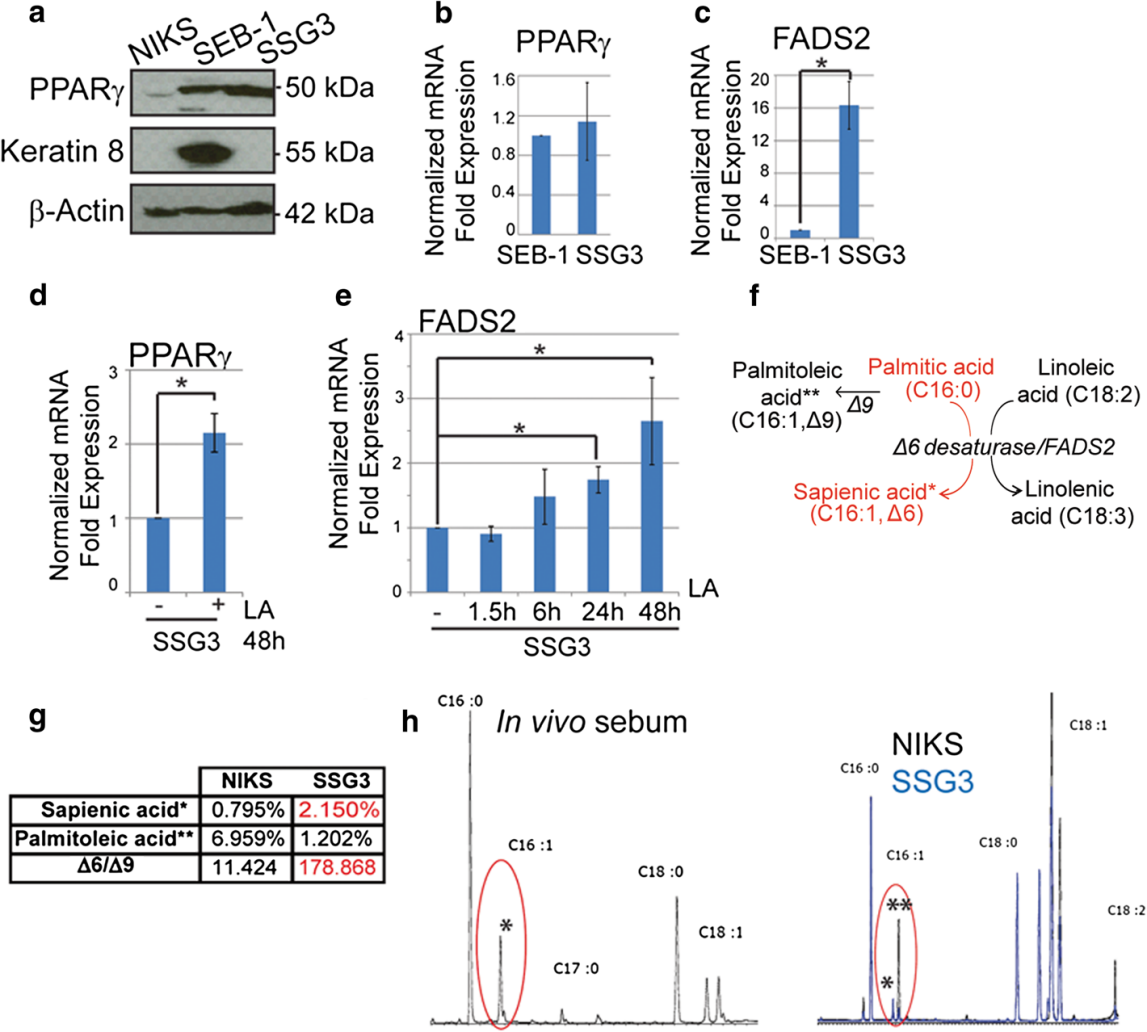
**Primary sebocytes isolated from scalp sebaceous glands can differentiate in vitro and produce sebum-characteristic lipids.** (**a**) SSG3 expresses PPARγ but not Keratin 8 in contrast to SEB-1. (**b-c**) Real-time PCR shows that *PPARγ* is equally expressed in SEB-1 and SSG3 whereas *FADS2* is more highly expressed in SSG3 cells than SEB-1. RNA from SEB-1 and SSG3 derived from the scalp explant at passage 3 were normalized to *GAPDH* expression. Data shown represent three independent experiments each performed in triplicate (mean +/−SD, n=3). * p-value <0.05 (unpaired two-tailed student’s *t* test). (**d**) Cells were cultivated for 48 h with or without 0.1 mM of linoleic acid (LA). Differentiation through LA activation is followed by an increase in *PPARγ* expression in SSG3 cells. * p-value <0.05 paired two-tailed student’s *t* test). (**e**) 24 h and 48 h of LA treatment induce a significant increase of *FADS2* expression in SSG3 cells. * p-value <0.05 (paired two-tailed student’s *t* test). (**f**) The Δ6 desaturase/FADS2 catalyzes the transformation of palmitic acid into sapienic acid. (**g**) Lipid analysis showing the percentage of Δ9 and Δ6 in the pellet of NIKS and SSG3 and the ratio Δ6/Δ9. (**h**) The sapienic acid (*) can be detected in SSG3 as in vivo sebum, whereas in NIKS, the palmitoleic acid (**) is the abundant lipid detected.

To verify that the cell cultures were indeed sebocytes, we examined the expression of known sebocyte markers. Immunofluorescence staining and immunoblot demonstrated that those cells homogeneously express peroxisome proliferator-activated receptor gamma (PPARγ) an adipogenic transcription factor expressed in differentiating sebocytes [26], in vitro (Figure 1d and Figure 2a*,* Scalp-derived Sebaceous Gland cells SSG3) and in vivo (Additional file 1: Figure S1a-b) but not in human keratinocytes (NIKS) [27]. Real-time PCR confirmed that primary SSG3 expressed a similar level of PPARγ as the immortalized sebocyte line SEB-1 [15] (Figure 2b). However, SEB-1 expresses Keratin 8, a protein associated with skin appendages tumors [28], whereas SSG3 cells do not express Keratin 8 (Figure 2a), akin to sebaceous gland in vivo [29]. Additionally, SSG3 cells express other markers of sebocytes such as Blimp1 and epithelial membrane antigen EMA/Muc1 (Additional file 1: Figure S1c, d and e). In agreement with recent reports [16,30], Blimp1 is expressed in the inner root sheath of the hair follicle and in terminally differentiated cells of the sebaceous glands in human scalp sections from which SSG3 cells were derived (Additional file 1: Figure S1c). All the results shown in scalp-derived sebocytes have been confirmed to be similar in the breast, chest and face derived-sebocytes (Additional file 1: Figure S1g). The only exception is the expression of Keratin 7, a marker of the undifferentiated sebocytes, detected at higher expression in protein lysates of the face-derived sebocytes compared to the scalp, the breast and the chest (Additional file 1: Figure S1f-g). The difference in Keratin 7 expression may depend on the location from which the cells derived (Additional file 1: Figure S1d). To conclude, we have established primary human sebocytes that express typical sebocyte markers and represent a good model for studying sebocyte function.

### *Primary sebocytes can differentiate* in vitro

To confirm that the primary human sebocytes are functional in vitro, we analyzed their ability to differentiate and produce human-specific lipids. The lipophilic dye Nile red can be used to stain terminally differentiating sebocytes [31] (Additional file 2: Figure S2a). Linoleic acid is an essential polyunsaturated fatty acid used for biosynthesis of arachidonic acid and other polyunsaturated fatty acids that can trigger the differentiation of sebocytes in vitro [32]. We therefore analyzed the cellular lipid distribution by Nile red after two days of linoleic acid treatment at physiological levels and show that SSG3 produce lipids in response to linoleic acid (Additional file 2: Figure S2b). Moreover, we detected cytosolic lipid droplets by electron microscopy in untreated cells (Additional file 2: Figure S2c) as well as an increase of lipid droplets with higher electron density after linoleic acid treatment (Additional file 2: Figure S2c”). Humans possess a unique *Δ6 desaturase/FADS2* gene [33] involved in linoleic acid metabolism and sebum production. *FADS2* is detectable mainly in differentiated sebocytes that have reached lipid synthesis capacity, providing a functional marker of activity and differentiation in sebocytes. We have found that *FADS2* is highly expressed in SSG3 cells compared to SEB-1 (Figure 2c). These results demonstrate that the SSG3 cells exhibit gene expression patterns characteristics of cells involved in sebocyte differentiation. Moreover, we found that the differentiation induced by linoleic acid treatment in SSG3 cells is followed by an increase in *PPARγ* at 48 h (Figure 2d) and an increase of *FADS2* after 24 h and 48 h of treatment when cells have reached a high level of cytoplasmic lipid production (Figure 2e).

To further confirm the presence of human specific lipids, gas chromatography of SSG3 cells was performed. We found differences in the composition of fatty acids, in particular, sapienic acid, predominantly found in sebum in vivo [33], and palmitoleic acid. They are synthesized by two desaturases, Δ6/FADS2 and Δ9 respectively [34] (Figure 2f). The desaturation in Δ6 position instead of Δ9 is specific to human sebum [34]. Sapienic acid is detected only in SSG3 cells (2.150%) compared to NIKS (0.795%). In contrast, palmitoleic acid is predominantly found in NIKS (6.959%) compared to SSG3 cells (1.202%) (Figure 2g and h). Next, to determine the functionality of SSG3 cells, we quantified the ratio of Δ6/Δ9 desaturase that is an index of sebocyte maturation and associated metabolic process [35]. We found that this ratio in SSG3 cells is largely superior to the NIKS (178.868 and 11.424 respectively) reflecting the functionality of the scalp-derived sebocytes (Figure 2g). The lipid analysis also revealed that only fatty acids with even-numbered carbon chains, a characteristic of in vivo sebum, are present in SSG3 (Figure 2h). We conclude that the primary human sebocyte cultures we have established not only express genes involved in sebum production and lipid synthesis but can also produce sebum-specific lipids. We next investigated the mechanism by which cellular differentiation and lipid production are regulated in primary human sebocytes.

### *TGFβ signaling is active in sebaceous gland* in vivo *and* in vitro

**Figure 3:**
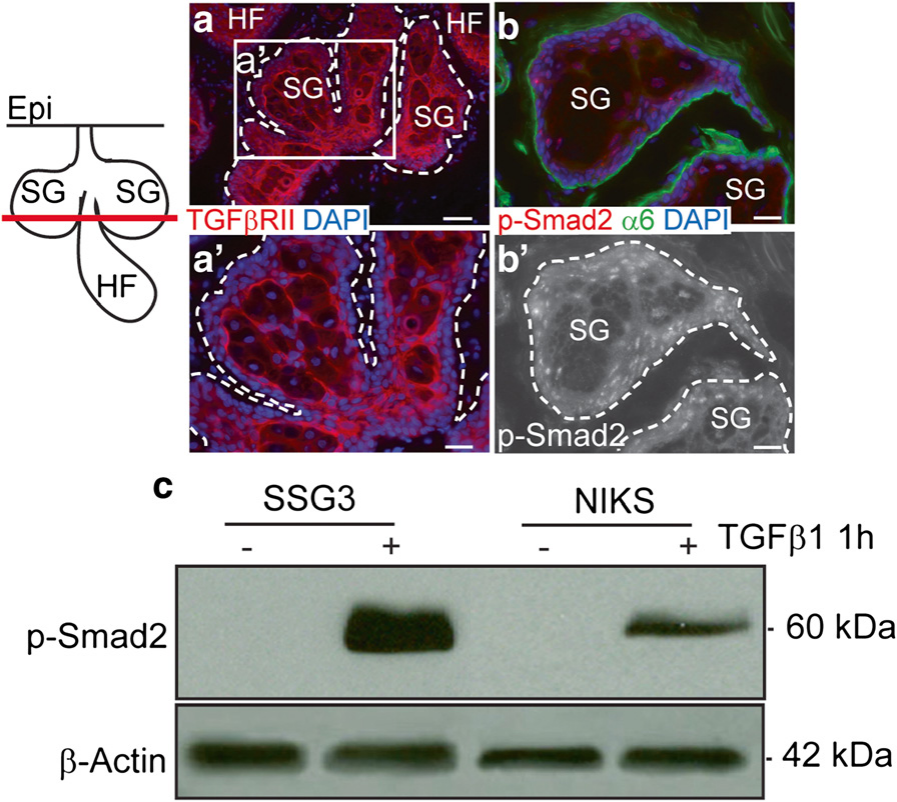
**TGFβ signaling is active in sebaceous gland in vivo and in vitro.** Sebaceous glands were sectioned in horizontal plane (red line in the diagram). (**a**) OCT sections of human scalp tissue stained with TGFβ RII (red) show expression of the receptor throughout the sebaceous gland with the exception of the differentiated cells in the center. Boxed area is magnified and shown to (**a’**). (**b**) TGFβ pathway is active in vivo as denoted by the expression of nuclear phosphorylated Smad2 (red). α6: α6-integrin stains in green the basal layer of the sebaceous gland. Scale bars, 50 μm (**a**), 20 μm (**a’**, **b**, **b’**). Abbreviations: Epi, Epidermis; HF, Hair Follicle; SG, Sebaceous Gland. (**c**) The indicated sebocyte cultures were treated with 5ng/ml of TGFβ1 ligand for one hour and whole cell extracts were examined by immunoblot to determine the activation of the TGFβ pathway.

A previous study using whole sebaceous gland explants treated with various cytokines, suggested TGFβ as a potential candidate for human sebocyte regulation [19]. TGFβ ligands bind to a bidimeric receptor complex composed of TGFβ RI and TGFβ RII to phosphorylate and activate receptor-bound Smad (Smad2/3) transcription factors enabling them to translocate into the nucleus and regulate TGFβ-responsive genes [36]. TGFβ RII is essential for the activation of the Smad2 pathway [23,37]. Therefore we analyzed the presence of TGFβ RII and the functionality of the pathway in vivo and in vitro by the presence of phosphorylated Smad2/3 as readout for TGFβ activation. Using immunofluorescence, we first verified that TGFβ RII is expressed throughout the sebaceous gland with the exception of the differentiated, lipid filled sebocytes present in the center of the gland (Figure 3a and 3a’). Further, we determined that the TGFβ pathway is active in the gland in vivo by detecting the expression of nuclear phosphorylated Smad2 in the undifferentiated and maturing sebocytes but not in terminally differentiated sebocytes present in the center of the gland (Figure 3b and 3b’). In vitro, Smad2 is phosphorylated in response to exogenously added recombinant TGFβ1 in SSG3 sebocytes, indicating the TGFβ pathway is intact and active in our in vitro system (Figure 3c).

### Effect of TGFβ signaling on sebocyte differentiation genes

**Figure 4:**
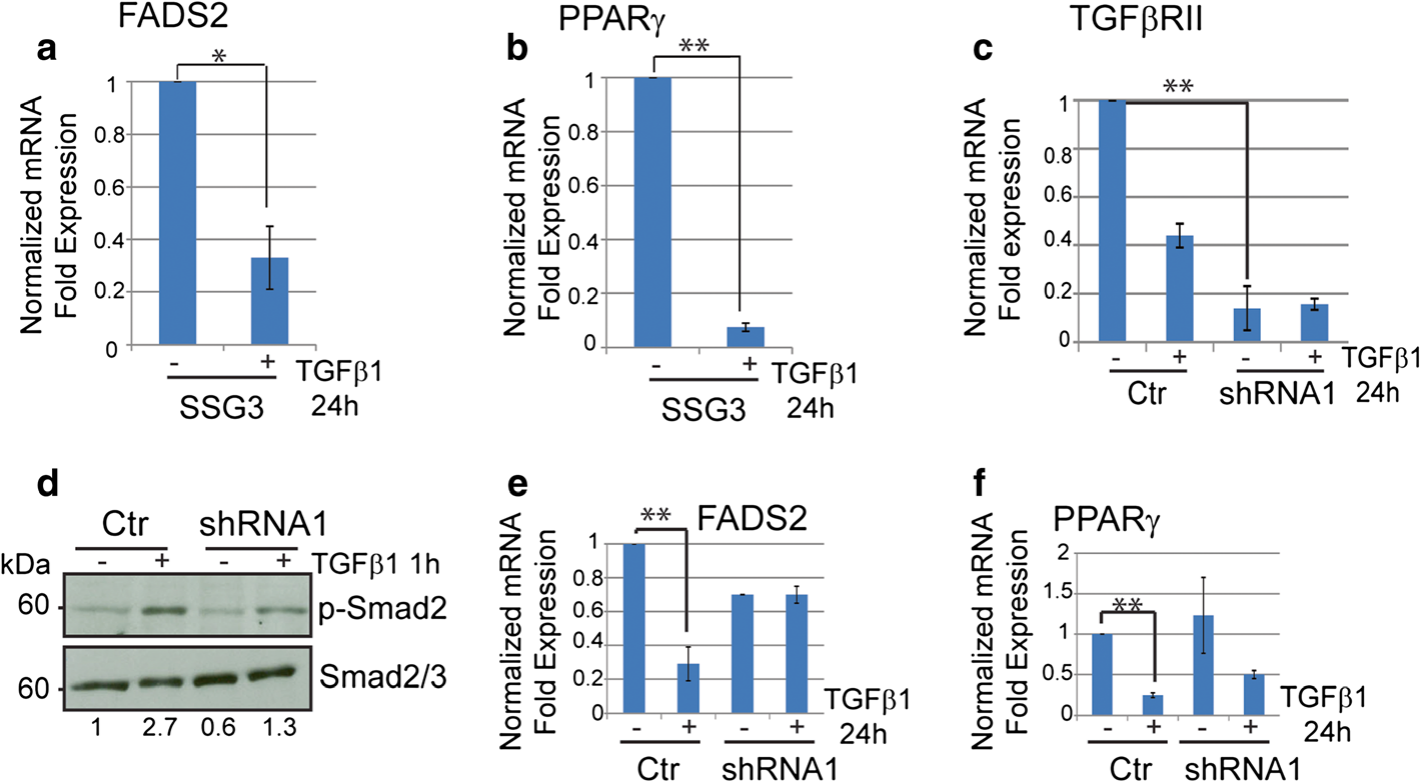
**TGFβ signaling triggered decreased expression of lipogenic genes through the TGFβ RII-Smad2 dependent pathway.** (**a, b**) SSG3 cells were treated with 5 ng/ml of TGFβ1 for 24 hours and used for qPCR. Data were normalized to *GAPDH* expression and relative expression determined using untreated cells as a reference. *FADS2* and *PPARγ* expression were found to be significantly downregulated in response to TGFβ1 treatment in SSG3 cells. (**c**) *TGFβ RII* expression in SSG3 cells expressing *TGFβ RII* shRNA1 and a control shRNA (Ctr) shows the efficiency of the knockdown. (**d**). Immunoblot confirms the decrease of p-Smad2 activity in shRNA expressing cells stimulated with TGFβ1 5ng/ml for 1 h. Values, noted below the immunoblot, represent the relative density quantified with ImageJ using the ratio p-Smad2/Smad2/3 from each condition. (**e, f**). Decrease of *FADS2* and *PPARγ* at the transcriptional level is mediated via canonical Smad signal transduction. The expression was normalized to control (Ctr) untreated. The significant decrease in *PPARγ* and *FADS2* genes in control SSG3 cells after treatment with TGFβ1, is not detected in *TGFβ RII*-deficient SSG3 cells. * p-value <0.05, ** p-value < 0.001 (paired two-tailed Student’s *t* test).

We next probed the effect of TGFβ signaling on their differentiation, by examining the expression of genes involved in lipogenesis upon treatment with TGFβ1. As shown in Figure 4a and b, when cells are stimulated with TGFβ1 for 24 h, the mRNA expression of *FADS2* and *PPARγ* are significantly decreased in SSG3 cells suggesting that TGFβ1 may prevent cell differentiation. Similar results were obtained in primary sebocytes derived from breast and face (Additional file 3: Figure S3), suggesting that the response to TGFβ is indicative of sebocytes in general and not due to the skin tissue type. To test if these effects are dependent on the canonical TGFβ pathway, we used shRNA to knockdown *TGFβ receptor II*, thus effectively inhibiting Smad2 phosphorylation [23]. *TGFβ RII* expression was similarly reduced in SSG3 cells using two independent TGFβ RII shRNA (Figure 4c). Phosphorylated-Smad2 was decreased in shRNA expressing cells compared to controls after TGFβ activation (Figure 4d), as expected. We also detected a decrease of *TGFβ RII* in control cells treated with TGFβ1 for 24 h (Figure 4c) reflecting the possible degradation of the receptor [38]. Moreover, the reduced *TGFβ RII* expression inhibited the ability of SSG3 cells to significantly decrease *FADS2* and *PPARγ* gene expression when cells are treated with TGFβ1 (Figure 4e and f). Our results indicate that the TGFβ pathway can directly control the expression of genes required for the differentiation of sebocytes.

**Figure 5:**
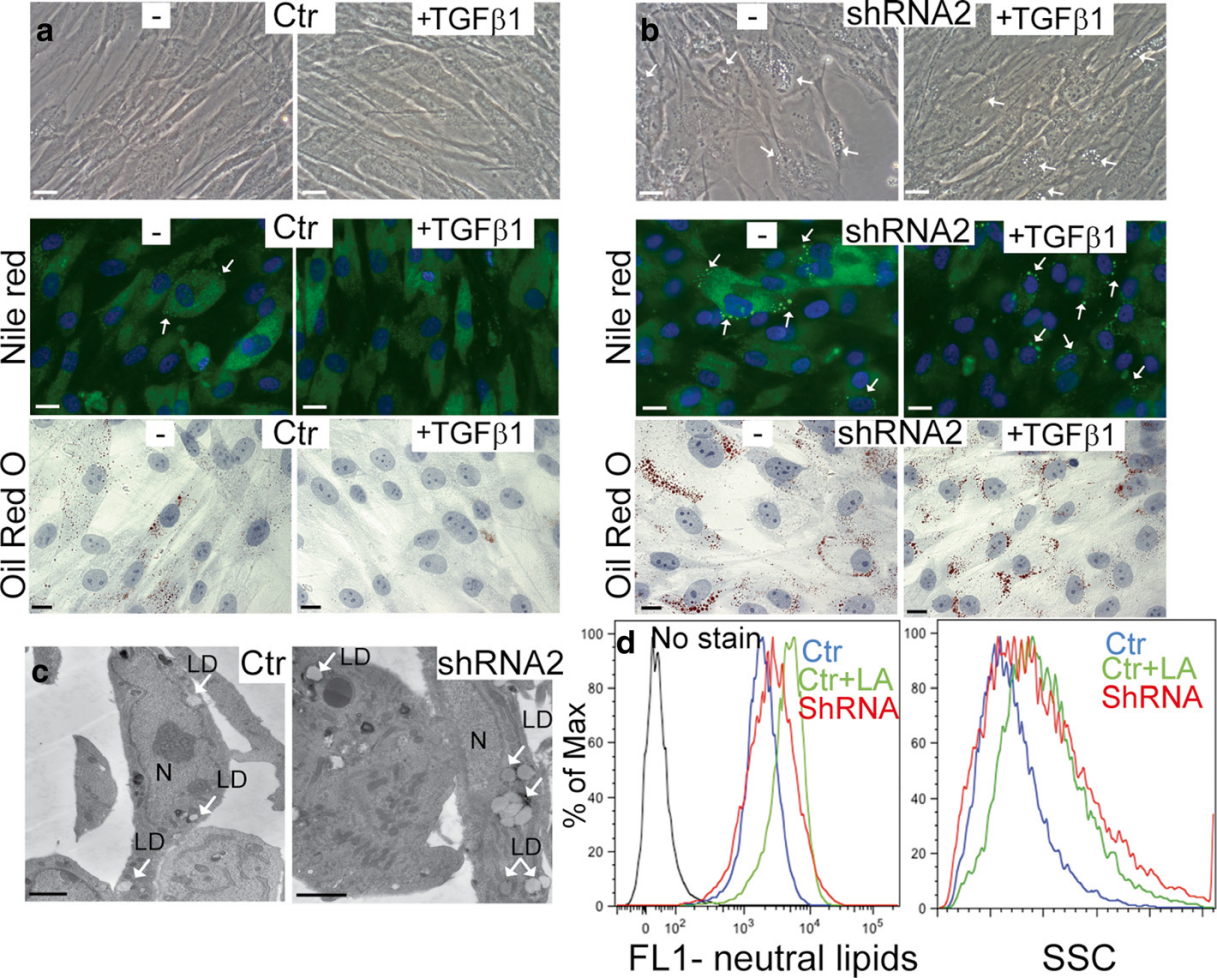
**Inhibition of TGFβ signaling induces lipogenesis in primary SSG3 cells.***(***a, b**) SSG3 cells stably expressing a shRNA against *TGFβ RII*, show accumulation of lipid droplets on brightfield images (scale bars, 20 μm), by Nile red (scale bars, 20 μm) and Oil red O stainings (scale bars, 10 μm). White arrows show the presence of multiple lipid droplets in the shRNA expressing cells compared to the control (Ctr). 24 h of TGFβ1 (5 ng/ml) treatment decreases the basal level of lipid production in control cells but does not affect cells expressing the *TGFβ RII* shRNA, mainly seen by Oil red O. (**c**) Electron microscopy showing the increase of lipid droplets in SSG3 cells (denoted by white arrows) expressing the shRNA compared to the control. Scale bars, 2 μm. LD, Lipid Droplets. N, Nucleus. (**d**) Flow cytometry of SSG3 cells expressing the shRNA labeled with Nile red. FL-1 measures the neutral lipids and SSC reflects the granularity of the cells. 10,000 cells have been acquired for each condition. As a positive control, SSG3 treated by 0.1 mM linoleic acid (LA) for 24 h show increase of fluorescence and granularity representing the lipid droplets. Note the increase of fluorescence and the increase of granularity in shRNA expressing *TGFβ RII* compared to the cells expressing a shRNA control. We obtained similar result with two different shRNA expressing *TGFβ RII* (Additional file 4: Figure S4).

Next we have determined how the inhibition of TGFβ signaling affects the functionality of SSG3 cells at a cellular level by analyzing the presence of cytoplasmic lipids in SSG3 shRNA expressing cells with reduced *TGFβ RII*. *TGFβ RII* depletion is associated with the increase of lipid inclusions positively stained with Nile red, Oil red O, and identified by electron microscopy compared to SSG3 cells expressing a shRNA control (Figure 5b and c and Additional file 4: Figure S4). The lipid droplets labeled with Nile red were analyzed by flow cytometry (Figure 5d). Similar to cells treated with linoleic acid, an increase in fluorescence and granularity (representing the lipid droplets) of the cells was detected in SSG3 *TGFβ RII* shRNA expressing cells compared to the shRNA control.

Additionally, we found that whereas TGFβ1 treatment has no effect on the lipid production in the shRNA cells (Figure 5b), it induces a decrease in lipid inclusion in SSG3 infected with a non-targeting shRNA control (Figure 5a). These results suggest that inhibition of *FADS2* and *PPARγ* at the transcriptional level is mediated via canonical Smad signal transduction. Together, our findings show that activation of the TGFβ signaling pathway down-regulates the expression of genes involved in the production of characteristic sebaceous lipids. We found that *TGFβ RII* gene, which is essential for the activation of the Smad2 pathway, limits lipid production in primary human sebocytes. These findings illustrate the role of TGFβ in maintaining human sebocytes in an undifferentiated state by inhibiting their differentiation and highlight the relevance of this pathway in human sebaceous gland biology.

## Discussion

Here we have developed a novel method of culturing human sebocytes without transformation and using a feeder layer-free culture system to examine the role of the TGFβ pathway in the control of differentiation. Primary sebaceous gland cells do not express Keratin 8 in contrast to previously immortalized sebocytes. Keratin 8 is not normally expressed in normal sebaceous gland in vivo [29] and our results indicate that the transformation process in the immortalized line has likely altered the expression of several fundamental cell markers. Moreover, we showed different responsiveness to linoleic acid and TGFβ1 treatment between the primary sebocytes and the immortalized cells (data not shown) suggesting that the cellular properties of those cells substantially differ.

Through our analysis, we have identified that certain markers of sebocytes are differentially expressed depending upon the location on the body (scalp, chest, face), and localization within the sebaceous gland. These results highlight the need for studies covering a range of patient ages to fully comprehend the regulation of the sebaceous glands. However, our work shows that the effect of TGFβ1 activation on sebocyte differentiation is similar in sebocytes derived from three areas (scalp, breast and face) suggesting the specificity of that effect is independent of location. Previous reports have largely focused on cells and glands derived from older adults and post-menopausal women [14-16]. While we have not identified differences in sex, the age of the individual from which the sebaceous gland is derived seems to be of significance. It is known that the sebaceous glands undergo dramatic changes over the course of one’s lifespan, with high sebum production occurring in infancy, a reduction during early childhood, followed by a steady increase through puberty into early adulthood. Using pediatric donors we ensured that the skin is not exposed to the hormonal changes that adult or old donor skin goes through. In the future it may be interesting to use our novel method to isolate sebocytes from old donors to examine the effect of age on TGFβ responsiveness in sebocytes.

We have begun to unravel one mechanism of differentiation of human sebaceous glands that culminates in sebum production. Our data suggest that TGFβ signaling maintains sebocytes in an undifferentiated state by decreasing the expression of *FADS2* and *PPARγ* thereby decreasing lipid accumulation through the TGFβ RII-Smad2 dependent pathway. The successful growth of these primary human sebocytes has important clinical application such as the possibility of designing new strategies of culturing engineered skin to enable and maintain the presence of sebaceous glands in skin grafts for burn victims [10,39]. In addition to cell autonomous regulators and signals inducing proliferation and maturation among sebaceous cells, the complex microenvironment surrounding the sebaceous gland might have a profound effect on homeostasis of the tissue. Molecular crosstalk between the dermis and the epithelial cells is crucial for the initiation and maintenance of the hair follicles [40]. It seems most likely that similar mechanisms of communication between sebocytes and the surrounding dermal tissue exist. For instance, in the mouse, TGFβ1 is known to be released by the inner root sheath of the hair follicle, thereby providing a means for a bidirectional interaction between the sebaceous gland and the hair follicle epithelium [20]. Similarly, in the dermis, human fibroblasts secrete TGFβ [41,42] which may then act on keratinocytes and sebocytes. Another component in the microenvironment that could also be part of this crosstalk are the arrector pili muscle cells recently shown to be controlled by bulge stem cells in mouse [43]. Being located in close proximity to the sebaceous gland, arrector pili muscles could help release sebum onto the skin surface [44].

Impairment of the skin barrier due to the deregulation of sebum production when associated with bacteria colonization and inflammation, can be the cause of serious skin conditions in people. For instance, hyperseborrhea combined with the presence of *Propionibacterium acnes* and inflammation can lead to acne vulgaris [4] and *Staphylococcus aureus* can aggravate atopic dermatitis [6]. Sebocytes can produce antimicrobial peptides such as defensin-1 and −2 upon exposure to *Propionibacterium acnes* or lipopolysaccharides [45,46] to prevent from bacteria colonization [47] and from an upregulation of sebum production [48]. Studies have revealed that TGFβ induces the expression of human β-defensin-2 in endothelial cells [49] and influences inflammatory response [50]. Therefore it will be interesting to further investigate the impact of TGFβ on immune responses in sebaceous gland and its implication in antimicrobial peptides secretion by sebocytes. With the novel isolation strategy we described here, different interactions with the microenvironment can now be investigated.

## Conclusions

By describing an innovative way to grow and successfully passage human primary sebocytes, we have overcome a major hurdle in the field of epithelial cell culture. We characterized the role of TGFβ signaling pathway in the inhibition of lipogenesis in these cells by showing that reduced expression of *TGFβ RII* increases lipid production. Our work, can not only improve our understanding of the physiology of the sebaceous gland in normal and pathological conditions [45] but also potentially expand this knowledge to other glands like eccrine and apocrine glands and use these cells to improve the quality of the skin grafts.

## Methods

### Cell Culture

The sebaceous gland populations were generated from human scalp (SSG3), face, chest and breast from both male and female donors. The skin samples were collected as a surgical waste with information provided regarding the age and sex of the donors with Institutional Review Board (IRB) approval at Cincinnati Children’s Hospital Medical Center. Cincinnati Children’s Hospital is a Pediatric Hospital that allowed us to collect samples from donors ranging 9 months old to 12 years old. The IRB determined that the research does not meet the regulatory criteria for research involving human subjects as there were no interaction with the donors and no identifiable private information. After treating the skin with dispase overnight at 4°C, intact sebaceous glands were isolated with microsurgical instruments under a dissecting microscope (Figure 1b). To mimic the microenvironment of the sebaceous gland, the explants were sandwiched between glass coverslips coated with human fibronectin (10 μg/ml, Millipore, Billerica, MA) (Figure 1d). The explants were cultivated in sebocyte medium as described [15] (DMEM/Ham’s F-12 (3:1), Epidermal Growth Factor (EGF 3 ng/ml, Austral Biologicals, San Ramon, CA), cholera toxin (1.2×10-10M, Sigma, St. Louis, MO), adenine (24 μg/ml, Sigma, St. Louis, MO), insulin (10 ng/ml Sigma), hydrocortisone (45.2 ng/ml, Sigma), FBS (2.5% Hyclone, San Jose, CA), antibiotic/antimycotic (100×, Invitrogen, Grand Island, NY). After 1–2 weeks of growth in culture, cellular outgrowth became apparent from the periphery of the gland lobules. The explants were removed and the isolated cells cultured on the fibronectin-coated coverslips.

### Western blotting

Proteins were separated by electrophoresis on 8-10% acrylamide gels, transferred to nitrocellulose membranes and subjected to immunoblotting. Membranes were blocked for one hour with 5% non-fat milk or 5% BSA in PBS containing 0.1% Tween-20. Primary antibodies were used at concentrations described below and HRP-coupled secondary antibodies were used at 1/2,000 in 5% non-fat milk. Immunoblots were developed using standard ECL (Amersham, Pittsburg, PA) and Luminata TM crescendo and classico (Millipore, Billerica, MA). Two-color immunoblot detection was performed using LI-COR Odyssey CL× (Biosciences, Lincoln, NE). Membranes were blocked in Odyssey blocking buffer (Li-Cor) and secondary antibodies conjugated to IRDye 680LT and 800CW were used (1/10,000; Li-Cor). Protein levels were quantified using the Odyssey Infrared Imaging System (Li-Cor).

### Retroviral Infection

To ablate *TGFβ RII* in SSG3 cells, we used shRNA vectors from the CCHMC Heart Institute lenti-shRNA library core (shRNA TGFβ RII #197031 and 194992 and a shRNA control). The human library was purchased from Sigma-Aldrich (MISSION shRNA). Lentivirus was produced by the Viral Vector Core at the Translational Core Laboratories, Cincinnati Children’s Hospital Research Foundation. Cells were grown to 80% confluency in 6-well plates before being infected with the lentivirus for 48 h. Infected cells were selected with 1 μg/ml puromycin (Sigma, St. Louis, MO) for 48 h. Following selection, *TGFβ RII* knock down cells were grown in regular media for 48 h before being activated with 5 ng/ml TGFβ1 for 24 h.

### Histology and Immunofluorescence

Human tissues were frozen unfixed in OCT compound (Tissue-Tek, Sakura, Torrance, CA) for cryosectioning. Immunostainings were performed as previously described [51].

### Antibodies

Primary antibodies against the following proteins were used at the dilution indicated: PPARγ (Santa-Cruz Biotechnology Inc., Santa Cruz, CA, H-100 1/250 for immunofluorescence, 1/500 for immunoblot), Blimp1 (Cell Signaling Technology, Beverly, MA, 1/500 for immunofluorescence, 1/1,000 for immunoblot), Fibronectin (Santa-Cruz Biotechnology Inc., Santa Cruz, CA, EP5 1/150), Muc1 (Millipore, Billerica, MA 1/500), cMyc (Cell Signaling Technology, Beverly, MA, 1/800 for immunofluorescence, 1/1,000 for immunoblot), TGFβ RII (Santa-Cruz Biotechnology Inc., Santa Cruz, CA, sc-220 1/1,000), p-Smad2 (Cell Signaling Technology, Beverly, MA, 1/100 for immunofluorescence, 1/1,000 for immunoblot), Smad2/3 (BD Biosciences, San Jose, CA, 1/500), α6 integrin (CD49f, BD Biosciences, San Jose, CA, 1/100), Keratin 8 (this antibody, developed by Dr. Brulet and Dr. Kemler, was obtained from the NICHD Developmental Studies Hybridoma Bank maintained by the University of Iowa, 1/1,000), β-actin (Sigma, St. Louis, MO, 1/2,000), Keratin 7 (Cell Signaling Technology, Beverly, MA, 1/1,000), 4^′^,6-diamidino-2-phenylindole (DAPI) was utilized as a marker of cell nuclei (Sigma Chemical Co., St. Louis, MO, 1/5,000). Secondary antibodies Alexa Fluor 488 or 555 (Molecular Probes, Grand Island, NY) were used at a dilution of 1/1,000. Fluorescence images were acquired with a fluorescent microscope AxioImager M1 (Zeiss, Oberkochen, Germany) and pictures were taken with an axioCam MRm camera (Zeiss, Oberkochen, Germany).

### Real-time PCR

Total RNA was isolated using a Qiagen Rneasy Mini Kit and used to produce cDNA (Maxima first strand cDNA synthesis kit, Fermentas, San Jose, CA). Reverse transcription (RT) reactions were diluted to 10 ng/μl and 1μl of each RT was used for real-time PCR. Real-time PCR was performed using the CFX96 real-time PCR System, CFX Manager Software and the SsoFast EvaGreen Supermix reagents (Biorad, Hercules, CA). All reactions were run in triplicate and analyzed using the ΔΔCT method with relative expression normalized to GAPDH. Primers used:

GAPDH-F: ACATCGCTCAGACACCATG, GAPDH-R: TGTAGTTGAGGTCAATGAAGGG

PPARγ-F: GAGCCCAAGTTTGAGTTTGC, PPARγ-R: GCAGGTTGTCTTGAATGTCTTC,

FADS2-F: TGTCTACAGAAAACCCAAGTGG, FADS2-R: TGTGGAAGATGTTAGGCTTGG,

TGFβ RII-F: CTGTGGATGACCTGGCTAAC, TGFβ RII-R: CATTTCCCAGAGCACCAGAG

### Lipogenesis assays

For Nile red staining, cells or OCT sections were fixed 10 minutes at room temperature in 4% formaldehyde. After 3 washes in 1XPBS, Nile red staining was performed with 0.1 μg/ml of Nile red (Sigma, St. Louis, MO) in 0.15 M NaCl for 15 minutes at room temperature. For Oil red O staining, cells were fixed 15 minutes in 10% formalin, wash with water for 10 minutes and 60% isopropanol before being stained with Oil red O (0.7% in 60% isopropanol) for 45 minutes. Cells were rinsed with 60% isopropanol and the nuclei stained with haematoxylin. To trigger differentiation of sebocytes in vitro, 0.1 mM linoleic acid (Sigma, St Louis, MO) was added directly to sebocyte media. To prepare cells for extraction of lipids, 2-3×10^7^ of cells were pelleted, washed with 1XPBS and lipids were preserved in the dark at −80°C under argon until analysis. The qualitative and quantitative composition of lipids in scalp-derived human sebocytes was determined using an Agilent 5973N Gas chromatograph/Mass spectrometer with a SPE cartridge (solid phase extraction) and was performed by Synelvia S.A.S (Labege, France).

### Nile Red analysis by FACS

Cells were cultured in 6-well plates at 80% confluence and infected with the lentivirus expressing the shRNAs as previously described. After puromycin selection for 48 h, cells were washed in 1X PBS and treated with working medium with or without Linoleic acid (0.1 mM) for 24 h. The cells were trypsinized, washed once with 1X PBS and neutral lipids were labeled with the fluorescent dye Nile red (1 μg/ml in PBS). 10,000 cells per sample were analyzed using a FACS Canto I equipped with a blue laser (488 nm excitation).

### Electron microscopy

Cells were grown at 80% confluency in sebocyte media and rinsed once with 0.175 M sodium cacodylate buffer. Cells were fixed in 3% glutaraldehyde/0.175 M cacodylate buffer (Electron Microscopy Sciences, Hatfield, PA) for 1 hour at 4°C. Dishes were washed twice with 0.175 M sodium cacodylate buffer. Cells were post fixed in 1% osmium tetroxide/cacodylate buffer for 1 hour at 4°C before being washed three times with 0.175 M sodium cacodylate buffer. After the final wash with 1.5 ml, cells were scraped and centrifuged for 5 min at 10,000 RPM. The cell pellet was then resuspended in 1 ml 1% agarose (Type IX ultra-low gelling tempt, Sigma) overnight at 4°C. The samples were then processed through a graded series of alcohols, infiltrated and embedded in LX-112 resin. After polymerization at 60°C for three days, ultrathin sections (100 nm) were cut using a Reichert-Jung Ultracut E microtome and counterstained in 2% aqueous uranyl acetate and Reynolds lead citrate. Images were taken with a transmission electron microscope (Hitachi H-6750) equipped with a digital camera (AMT 2k×2K tem CCD).

### Statistics

Data are expressed as means +/− SD. Comparison between two cell types was performed using unpaired two-tailed student’s *t* test. Paired two-tailed student’s *t* test was used when we compared the effect of a treatment on the same cell type. p<0.05 was considered significant.

## Competing interests

GG has no conflict of interest. JD, CB and LB are employed by L’Oreal and have conflict of interests.

## Pre-publication history

The pre-publication history for this paper can be accessed here:

http://www.biomedcentral.com/1471-5945/13/2/prepub

## Supplementary Material

Additional file 1: Figure S1 Primary human sebocytes derived from scalp, breast, chest and face tissues express typical sebocyte markers. (a) Hematoxylin and Eosin staining of the scalp sample. Scale bar, 50 μm. (b) Immunofluorescence staining showed that PPARγ (red) is expressed in human sebaceous glands from the scalp explant at the periphery stained with α6-integrin (green) and at the center of the gland. Scale bar, 50 μm. Boxed area is magnified and shown to (b’). (c) Blimp1 (red) expression is mostly found in the differentiated cells of the sebaceous gland and in the inner root sheath of the hair follicle. α6-integrin (green) marked the basal layer of the gland. (d) Keratin 7 (red) expression varies depending on the location of the gland (scalp, breast and chest) as shown by immunofluorescence. (e-g) Sebocytes derived from the scalp, breast, chest and face explants expressed sebocytes markers by two-color immunoblot (Blimp1, c-Myc, Muc1, PPARγ and K7). SSG4 represents primary sebocytes derived from a four year old-scalp sample. Scale bars, 50 μm (b), 50 μm (c and d). Abbreviations: SG, Sebaceous Gland; HF, Hair Follicle; α6, α6-integrin; K7, Keratin 7. Click here for file

Additional file 2: Figure S2 Primary sebocytes can differentiate in vitro. (a) Human scalp sections showing evidence of lipid accumulation (Nile red stain). Scale bar, 50 μm (b) SSG3 cells derived from the scalp explants were treated with 0.1 mM linoleic acid (LA) for 48 h to differentiate the cells and stained with Nile red to detect lipids. Images were taken with the same exposure time in untreated and linoleic acid-treated conditions. Brightfield pictures showed accumulation of cytoplasmic lipid droplets after linoleic acid treatment as denoted by the black arrows. Scale bars, 50 μm (c) Electron microscopy showing cytoplasmic lipid droplets in untreated sebocytes SSG3 derived from the scalp explants. Scale bar, 20 μm. Boxed area is magnified and shown to (c’) scale bar, 500 nm. (c”) After linoleic acid treatment increased high-electron density lipid droplets are detected in SSG3 cells and magnified in c”’. Scale bars for c” and c”’ are 2 μm. Abbreviations: HF, Hair Follicle. SG, Sebaceous Gland. LD, Lipid Droplets. N, Nucleus. Mi, Mitochondria. RER, Rough Endoplasmic Reticulum . SER, Smooth Endoplasmic Reticulum. Click here for file

Additional file 3: Figure S3 TGFβ signaling triggered decreased expression of lipogenic genes in breast and face-derived sebocytes. RNA was isolated from sebocytes-derived from breast and face untreated or treated with 5 ng/ml of TGFβ1 for 24 h and used for real-time PCR. Two experiments were performed and all qPCR reactions were performed in triplicate. Data were normalized to *GAPDH* expression for each cell population and changes in relative expression were determined using untreated cells as a reference point. (a) *FADS2* and (b) *PPARγ* expression was found to be decreased significantly in response to TGFβ1 treatment as shown in scalp-derived sebocytes (Figure 4a-b) suggesting that the inhibitory effect of TGFβ is not due to the skin tissue type. *p-value<0.05 (paired two-tailed Student’s *t* test). Click here for file

Additional file 4: Figure S4 Inhibition of TGFβ signaling induces lipogenesis in primary SSG3 cells. (a) SSG3 cells, stably expressing a shRNA against *TGFβ RII* (shRNA1), show accumulation of lipid droplets on brightfield image (white arrows) and by Nile red staining (shown in green) compared to cells infected with shRNA control. Scale bars, 20 μm. (b-c), Electron microscopy showing the increase of lipid droplets in SSG3 cells (denoted by white arrows) expressing the shRNA against *TGFβ RII* (shRNA2) compared to the control. Myelin figures, which indicate lipids synthesis, are detected in SSG3 cells expressing the shRNA. Abbreviations: N, nucleus. LD, Lipid Droplets. Scale bars for b and c are 2 μm and 500 nm for c’. Click here for file
