# Supplementary figures and images for: TGFβ signaling regulates lipogenesis in human sebaceous glands cells
Source: BMC Dermatol. 2013 Jan 23;13:2. doi: 10.1186/1471-5945-13-2 (PMC3610216; doi:10.1186/1471-5945-13-2)

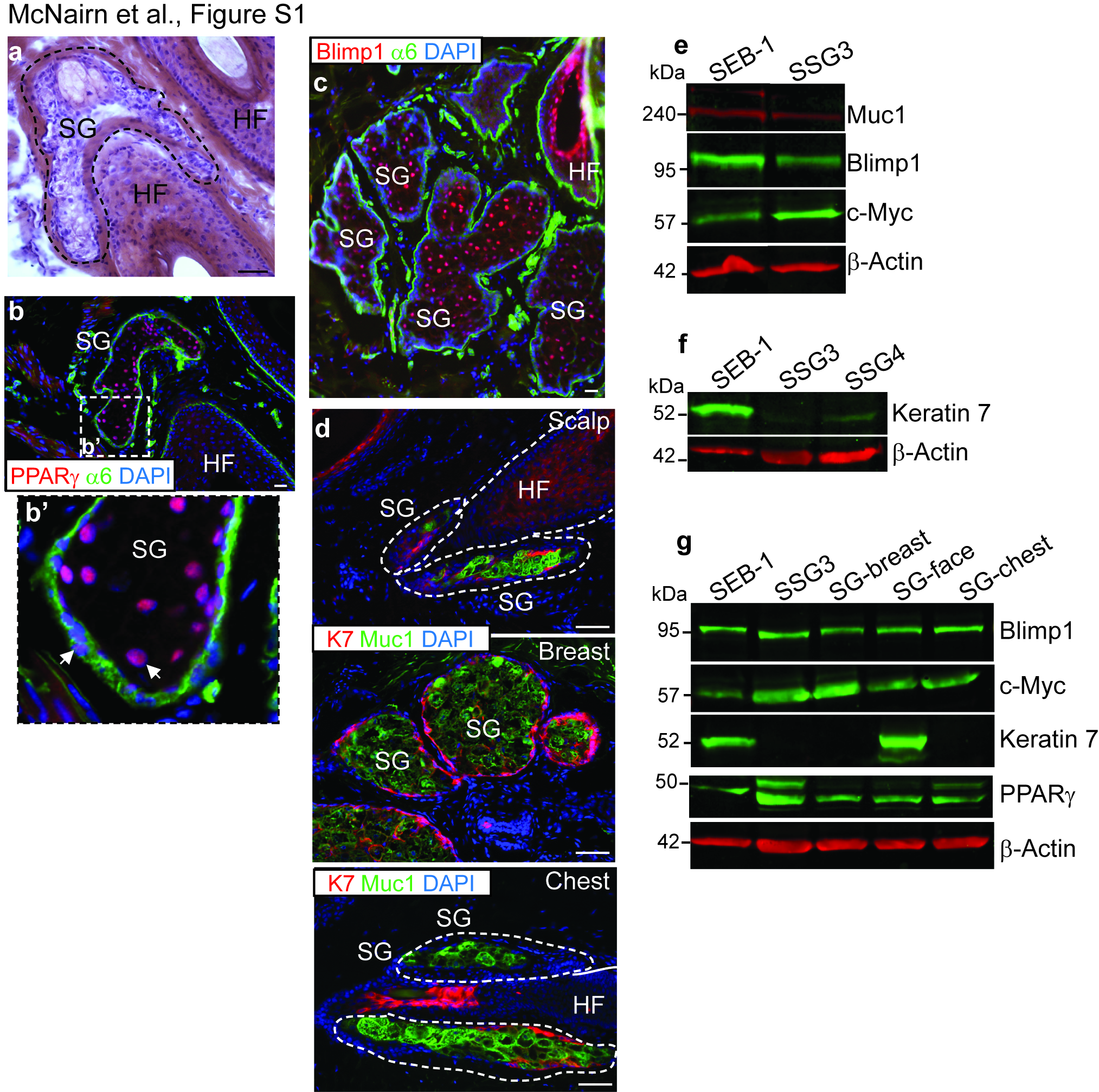

Supplement: Additional file 1: Figure S1 — Primary human sebocytes derived from scalp, breast, chest and face tissues express typical sebocyte markers. (a) Hematoxylin and Eosin staining of the scalp sample. Scale bar, 50 μm. (b) Immunofluorescence staining showed that PPARγ (red) is expressed in human sebaceous glands from the scalp explant at the periphery stained with α6-integrin (green) and at the center of the gland. Scale bar, 50 μm. Boxed area is magnified and shown to (b’). (c) Blimp1 (red) expression is mostly found in the differentiated cells of the sebaceous gland and in the inner root sheath of the hair follicle. α6-integrin (green) marked the basal layer of the gland. (d) Keratin 7 (red) expression varies depending on the location of the gland (scalp, breast and chest) as shown by immunofluorescence. (e-g) Sebocytes derived from the scalp, breast, chest and face explants expressed sebocytes markers by two-color immunoblot (Blimp1, c-Myc, Muc1, PPARγ and K7). SSG4 represents primary sebocytes derived from a four year old-scalp sample. Scale bars, 50 μm (b), 50 μm (c and d). Abbreviations: SG, Sebaceous Gland; HF, Hair Follicle; α6, α6-integrin; K7, Keratin 7. [file 1471-5945-13-2-S1.tiff]

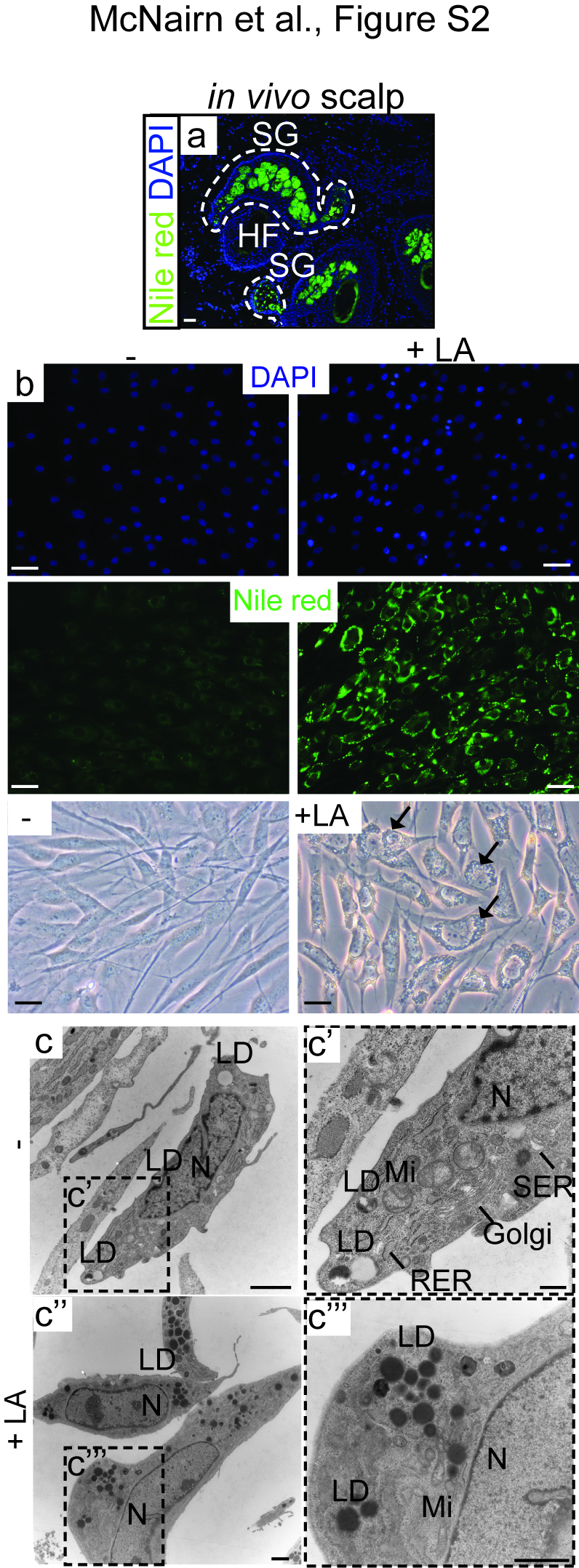

Supplement: Additional file 2: Figure S2 — Primary sebocytes can differentiate in vitro. (a) Human scalp sections showing evidence of lipid accumulation (Nile red stain). Scale bar, 50 μm (b) SSG3 cells derived from the scalp explants were treated with 0.1 mM linoleic acid (LA) for 48 h to differentiate the cells and stained with Nile red to detect lipids. Images were taken with the same exposure time in untreated and linoleic acid-treated conditions. Brightfield pictures showed accumulation of cytoplasmic lipid droplets after linoleic acid treatment as denoted by the black arrows. Scale bars, 50 μm (c) Electron microscopy showing cytoplasmic lipid droplets in untreated sebocytes SSG3 derived from the scalp explants. Scale bar, 20 μm. Boxed area is magnified and shown to (c’) scale bar, 500 nm. (c”) After linoleic acid treatment increased high-electron density lipid droplets are detected in SSG3 cells and magnified in c”’. Scale bars for c” and c”’ are 2 μm. Abbreviations: HF, Hair Follicle. SG, Sebaceous Gland. LD, Lipid Droplets. N, Nucleus. Mi, Mitochondria. RER, Rough Endoplasmic Reticulum . SER, Smooth Endoplasmic Reticulum. [file 1471-5945-13-2-S2.tiff]

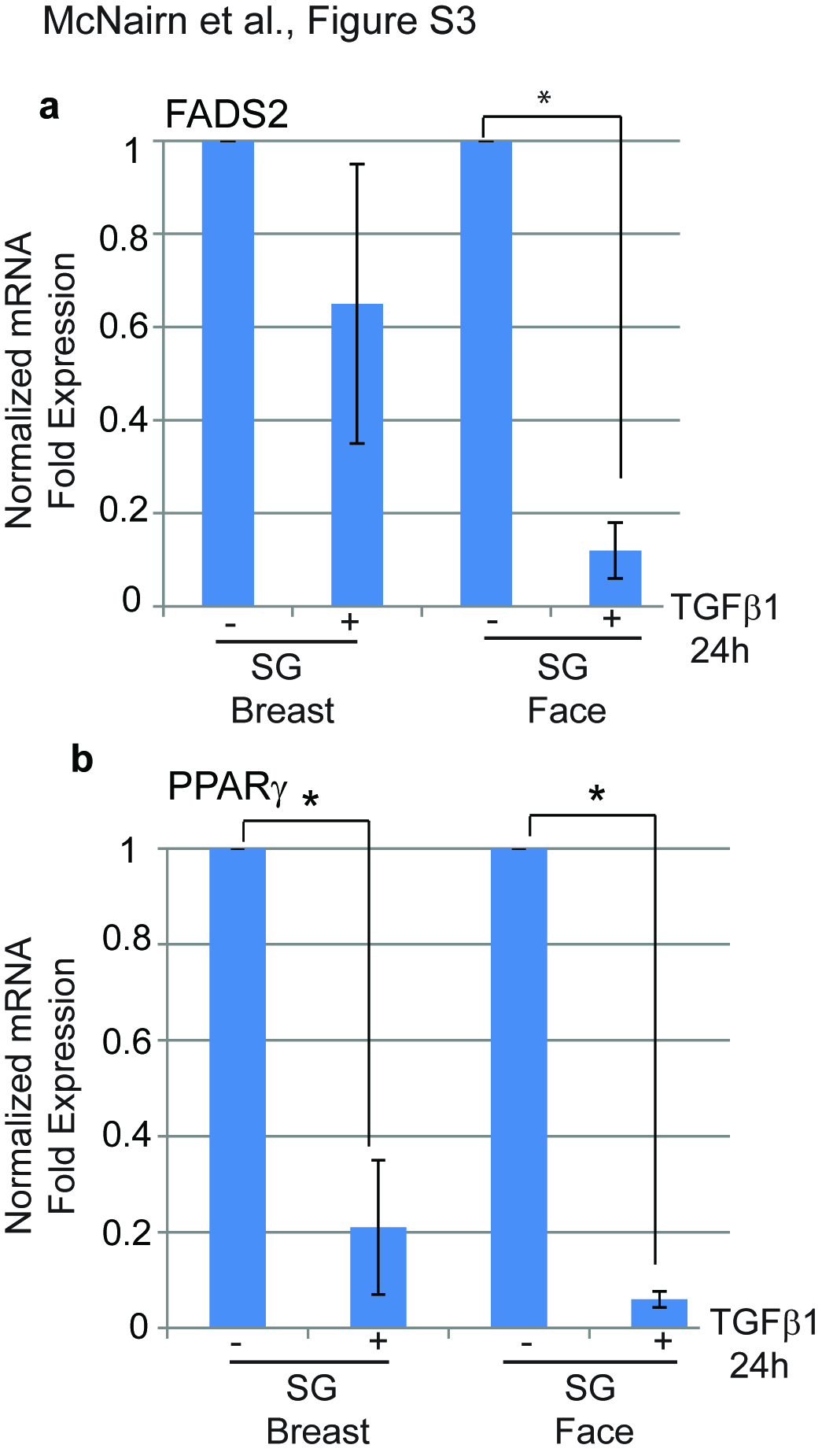

Supplement: Additional file 3: Figure S3 — TGFβ signaling triggered decreased expression of lipogenic genes in breast and face-derived sebocytes. RNA was isolated from sebocytes-derived from breast and face untreated or treated with 5 ng/ml of TGFβ1 for 24 h and used for real-time PCR. Two experiments were performed and all qPCR reactions were performed in triplicate. Data were normalized to GAPDH expression for each cell population and changes in relative expression were determined using untreated cells as a reference point. (a) FADS2 and (b) PPARγ expression was found to be decreased significantly in response to TGFβ1 treatment as shown in scalp-derived sebocytes (Figure 4a-b) suggesting that the inhibitory effect of TGFβ is not due to the skin tissue type. *p-value<0.05 (paired two-tailed Student’s t test). [file 1471-5945-13-2-S3.tiff]

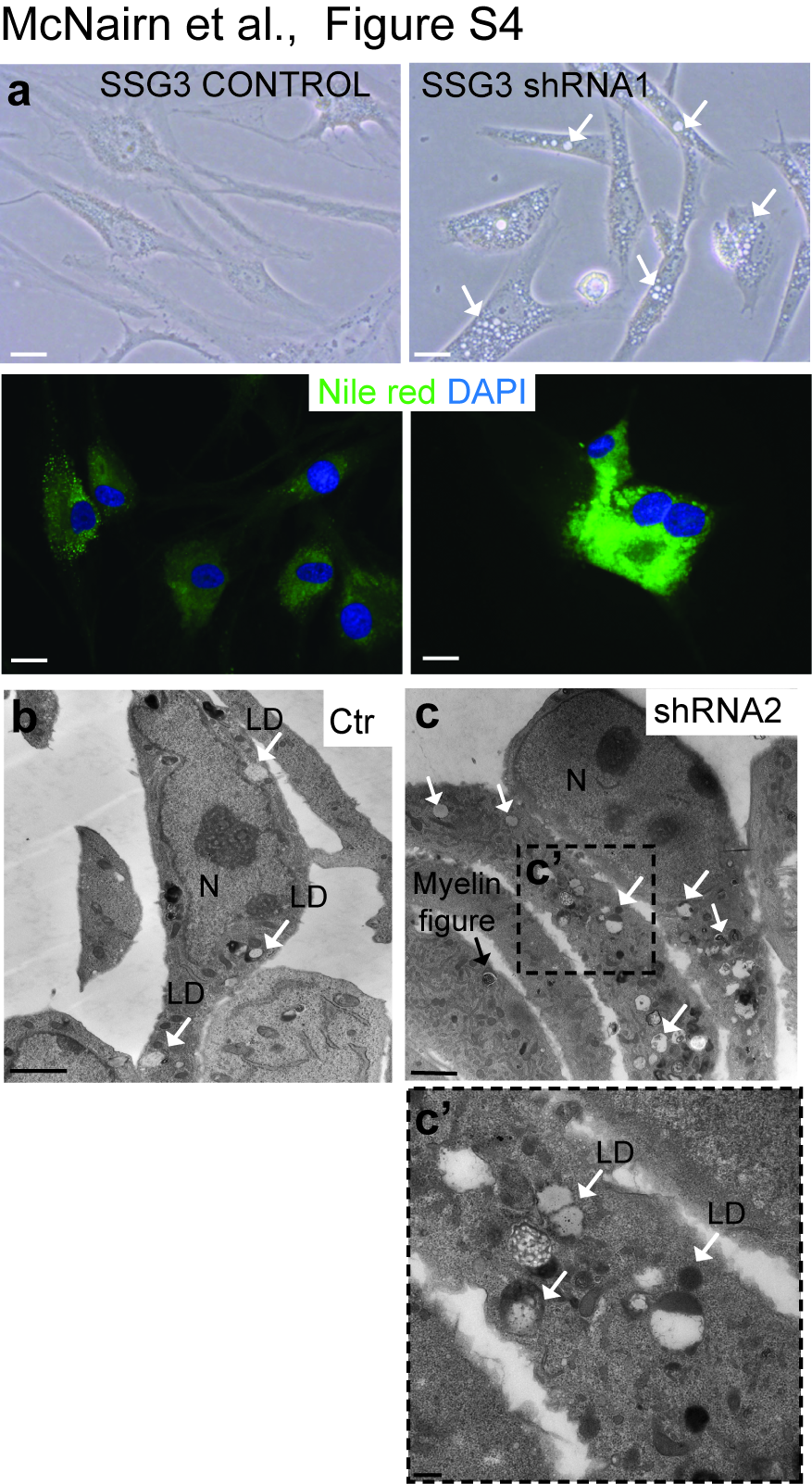

Supplement: Additional file 4: Figure S4 — Inhibition of TGFβ signaling induces lipogenesis in primary SSG3 cells. (a) SSG3 cells, stably expressing a shRNA against TGFβ RII (shRNA1), show accumulation of lipid droplets on brightfield image (white arrows) and by Nile red staining (shown in green) compared to cells infected with shRNA control. Scale bars, 20 μm. (b-c), Electron microscopy showing the increase of lipid droplets in SSG3 cells (denoted by white arrows) expressing the shRNA against TGFβ RII (shRNA2) compared to the control. Myelin figures, which indicate lipids synthesis, are detected in SSG3 cells expressing the shRNA. Abbreviations: N, nucleus. LD, Lipid Droplets. Scale bars for b and c are 2 μm and 500 nm for c’. [file 1471-5945-13-2-S4.tiff]
